# Scale-Up Transformed Shoot and Hairy Root Cultures of *Salvia bulleyana* in Bioreactor Systems: Process Optimization and Metabolite Productivity

**DOI:** 10.3390/molecules31162856

**Published:** 2026-08-15

**Authors:** Marta Krzemińska, Aleksandra Owczarek-Januszkiewicz, Monika A. Olszewska, Izabela Grzegorczyk-Karolak

**Affiliations:** 1 Department of Biology and Pharmaceutical Botany, Medical University of Lodz, Muszynskiego 1, 90-151 Lodz, Poland; izabela.grzegorczyk@umed.lodz.pl; 2 Department of Pharmacognosy, Medical University of Lodz, Muszynskiego 1, 90-151 Lodz, Poland; aleksandra.owczarek@umed.lodz.pl (A.O.-J.); monika.olszewska@umed.lodz.pl (M.A.O.)

**Keywords:** elicitation, methyl jasmonate, nutrient sprinkle bioreactor, polyphenol productivity, rosmarinic acid, temporary immersion bioreactor, transformed culture

## Abstract

To ensure large-scale production of plant-derived polyphenols, efficient and scalable in vitro culture systems are needed that can enable high biomass accumulation and secondary metabolite biosynthesis. The present study evaluates the suitability of transformed *Salvia bulleyana* shoot and hairy root cultures for biomass growth and phenolic compound production in different bioreactor systems, including two temporary immersion systems (PlantForm™ and RITA^®^) and a nutrient sprinkle bioreactor (NSB). A pronounced system- and organ-specific response was observed. In transformed shoot cultures, the RITA^®^ bioreactor promoted high growth and volumetric productivity, although metabolite accumulation was lowered by partial hyperhydricity. In contrast, the PlantForm™ system provided more stable morphogenesis and higher polyphenol content, although less effective growth. In hairy root cultures, the NSB proved to be the most effective system, combining high biomass productivity with greater phenolic compound accumulation; total polyphenol content reached 65.1 mg/g DW, with rosmarinic acid (RA) constituting up to 78% of total phenolics. The highest volumetric productivity was achieved in NSB-grown hairy roots, reaching 837.9 mg/L total polyphenols and 651.3 mg/L RA. Secondary metabolism was further enhanced by elicitation with 100 μm methyl jasmonate applied under optimized bioreactor conditions. In transformed shoot cultures, elicitation increased RA accumulation by 63%, resulting in final productivity of 1164 mg/L RA, and 1250 mg/L total polyphenols. In hairy root cultures, RA accumulation increased by 44%, leading to final productivity of 795 mg/L RA and 933 mg/L total polyphenols. Our findings indicate that the balance between biomass growth and secondary metabolism in *S. bulleyana* cultures is determined by bioreactor configuration. The nutrient sprinkle bioreactor represents a highly effective platform for phenolic acid production in hairy root cultures, whereas temporary immersion systems can be successfully applied for transformed shoot cultivation. The combination of tailored bioreactor strategies with methyl jasmonate elicitation provides a promising approach for scalable production of high-value phenolic compounds in plant in vitro culture systems.

## 1. Introduction

The increasing demand for natural bioactive compounds has intensified interest in plant-derived polyphenols with well-documented antioxidant, anti-inflammatory, and anticancer properties [1,2]. Among these phenolic compounds, rosmarinic acid (RA) and its derivatives represent a particularly important group with widespread application in the pharmaceutical, cosmetic, and food industries [3]. The growing interest in the health-promoting properties and potential applications of RA has increased the demand for high-quality, reliable sources. Hence, plant in vitro culture has emerged as a promising alternative to conventional cultivation systems, offering controlled and reproducible production independent of environmental variability, together with the possibility of enhancing metabolite accumulation through targeted biotechnological strategies [4,5].

Within the genus *Salvia*, numerous species are recognized as valuable sources of RA and salvianolic acid derivatives. Among them, *Salvia bulleyana* Diels, traditionally used in Chinese medicine as a substitute for *S. miltiorrhiza*, has attracted increasing attention due to its high content of bioactive polyphenols [6,7]. Previous studies demonstrated that transformed *S. bulleyana* shoot and hairy root cultures efficiently produce phenolic compounds, frequently at levels exceeding those observed in field-grown plants [8,9]. In particular, hairy root cultures induced by *Rhizobium rhizogenes* are considered highly suitable for secondary metabolite production because of their genetic stability, rapid growth, and high biosynthetic capacity [10]. The system also exhibits strong metabolic responsiveness, with phenolic acid biosynthesis being further stimulated by elicitation with methyl jasmonate [8,11].

Despite its considerable biosynthetic potential, the broader industrial application of plant in vitro culture is limited by issues associated with scalability, process reproducibility, and cultivation efficiency. Consequently, increasing attention has been directed toward the development of bioreactor-based cultivation systems, which can enable improved control of culture conditions and enhanced biomass and metabolite productivity [12,13]. Among these approaches, temporary immersion systems (TIS) are regarded as particularly advantageous for differentiated plant tissues; such approaches can improve aeration and reduce physiological stress by ensuring periodic contact with liquid medium while preventing continuous submergence [14,15,16]. The system has been found to facilitate enhanced biomass growth and secondary metabolite accumulation in shoots of a few species, including *Stevia rebaudiana*, *Salvia viridis*, and *Lycium barbarum* [17,18,19].

Alternatively, culture can be performed in nutrient sprinkle bioreactors, in which plant material is intermittently supplied with culture medium in the form of droplets or mist. Such systems combine efficient nutrient delivery with high oxygen availability while minimizing shear stress, making them particularly suitable for sensitive tissues such as hairy roots [20,21,22]. They have been found to be effective for enhancing RA accumulation in *Rindera graeca* cultures [23].

While evidence exists regarding the potential of temporary immersion and nutrient sprinkle systems, comparative studies evaluating their suitability for specific culture types and plant species remain limited. In particular, the scale-up of *S. bulleyana* cultures under controlled bioreactor conditions has not yet been investigated, despite their considerable potential as a source of RA and related polyphenols.

Therefore, the aim of the present study was to evaluate the suitability of selected bioreactor systems for the scale-up cultivation of transformed shoot and hairy root cultures of *S. bulleyana*. Specifically, it compares two temporary immersion systems (PlantForm™ and RITA^®^) and a nutrient sprinkle bioreactor (NSB) with respect to biomass growth, polyphenol accumulation, and volumetric productivity, and investigates the effects of immersion regimen and methyl jasmonate elicitation on secondary metabolite production.

## 2. Results

### 2.1. Transformed Shoot Culture

Transformed shoots of *S. bulleyana* were cultivated in two temporary immersion systems, PlantForm™ and RITA^®^, under identical culture conditions (MS medium supplemented with 0.1 mg/L IAA and 1.0 mg/L BAPR; immersion regime: 3 min every 90 min). After five weeks of cultivation, morphological parameters, biomass accumulation, and multiplication efficiency were evaluated.

**Figure 1:**
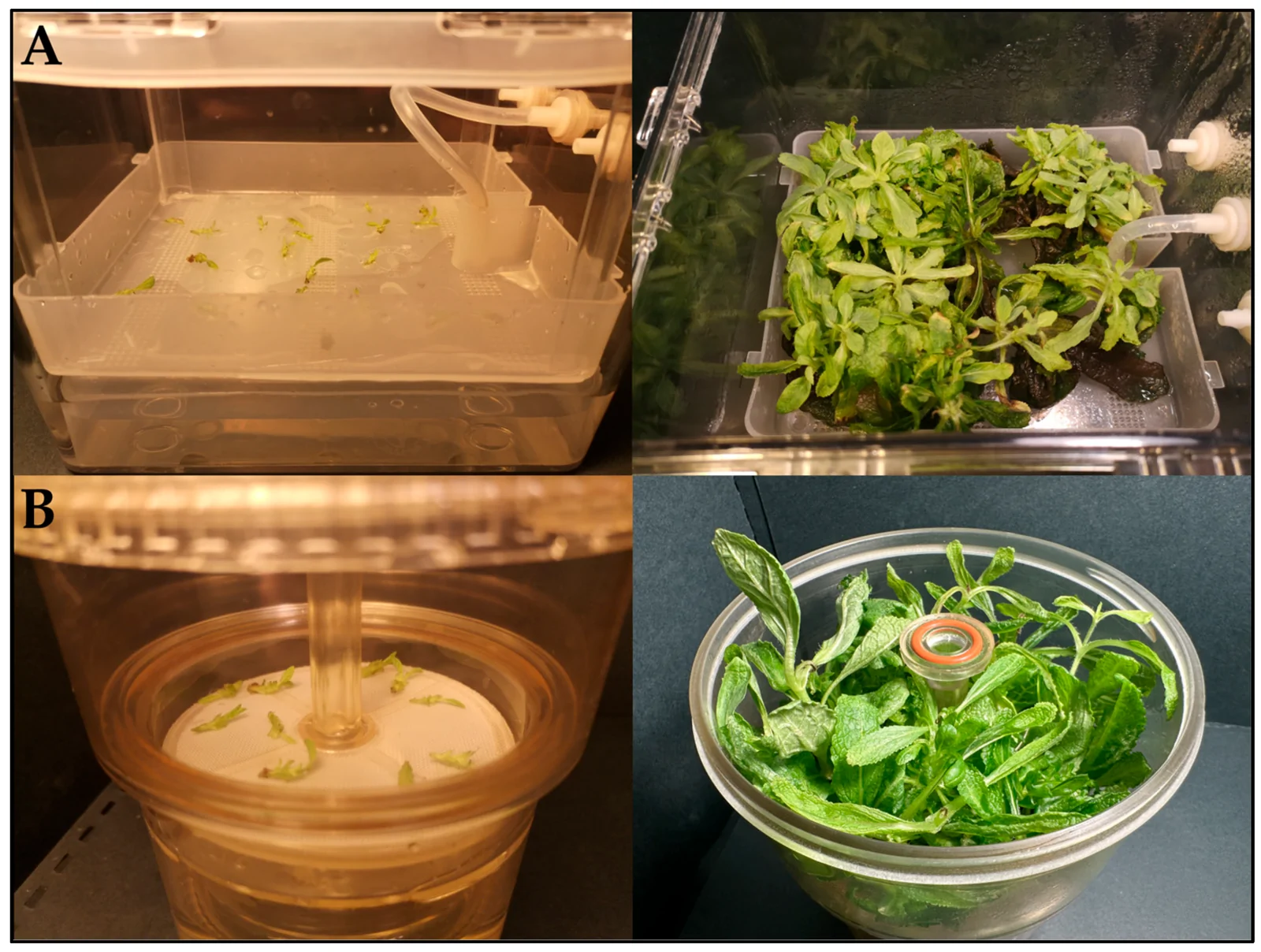
Transformed shoot culture of *S. bulleyana* cultivated in temporary immersion bioreactors: (**A**) PlantForm™ (inoculum, culture after 5 weeks); (**B**) RITA (inoculum, culture after 5 weeks).

Cultures maintained in the RITA^®^ bioreactor exhibited some morphological alterations associated with hyperhydricity, including tissue fragility, and translucency of leaves and stems (Figure 1). Despite these abnormalities, the multiplication rate reached 7.3 (Table 1). Most newly formed structures (93%) were buds (≤0.5 cm in height); these predominantly originated from the organogenic callus located at the base of explants or on hyperhydrated leaf tissues.

In the RITA^®^ system, *S. bulleyana* regenerated shoots reached a mean length of 1.25 cm, while the main shoots attained 4.08 cm (Table 1). Sporadic rhizogenesis was also observed, with roots reaching a mean length of 0.89 cm. Biomass accumulation was substantial, with fresh weight reaching approximately 47.6 g, corresponding to a growth index of 84.4, while the dry weight growth index reached 76.4 (Table 1).

In contrast, transformed shoots cultivated in the PlantForm™ bioreactor maintained predominantly normal morphology. Hyperhydricity was limited to basal shoot regions directly exposed to the liquid medium and became evident only in later stages of cultivation, where tissues showed translucency and progressive darkening (Figure 1).

The multiplication rate in the PlantForm™ system (5.26) was approximately 28% lower than in the RITA^®^ bioreactor. The mean shoot length was 1.15 cm; however, similarly to the RITA^®^ system, many regenerated structures were buds. The mean length of the main shoots (2.55 cm) was nearly 50% less than in the RITA^®^ culture (Table 1).

Although the PlantForm™ bioreactor demonstrated higher absolute biomass accumulation (69.2 g FW and 6.67 g DW) than the RITA^®^ system (Table 1), this difference resulted primarily from the larger initial inoculum and medium volume applied in the former. When normalized to the initial inoculum, the growth indices in the PlantForm™ bioreactor were 61.0 for fresh weight and 64.4 for dry weight. These results suggest that, despite lower total biomass yield, the RITA^®^ system provided more favorable conditions for shoot growth dynamics and biomass increment relative to the inoculum and medium volume.

The type of temporary immersion system significantly influenced secondary metabolite biosynthesis in the transformed *S. bulleyana* shoots.

**Figure 2:**
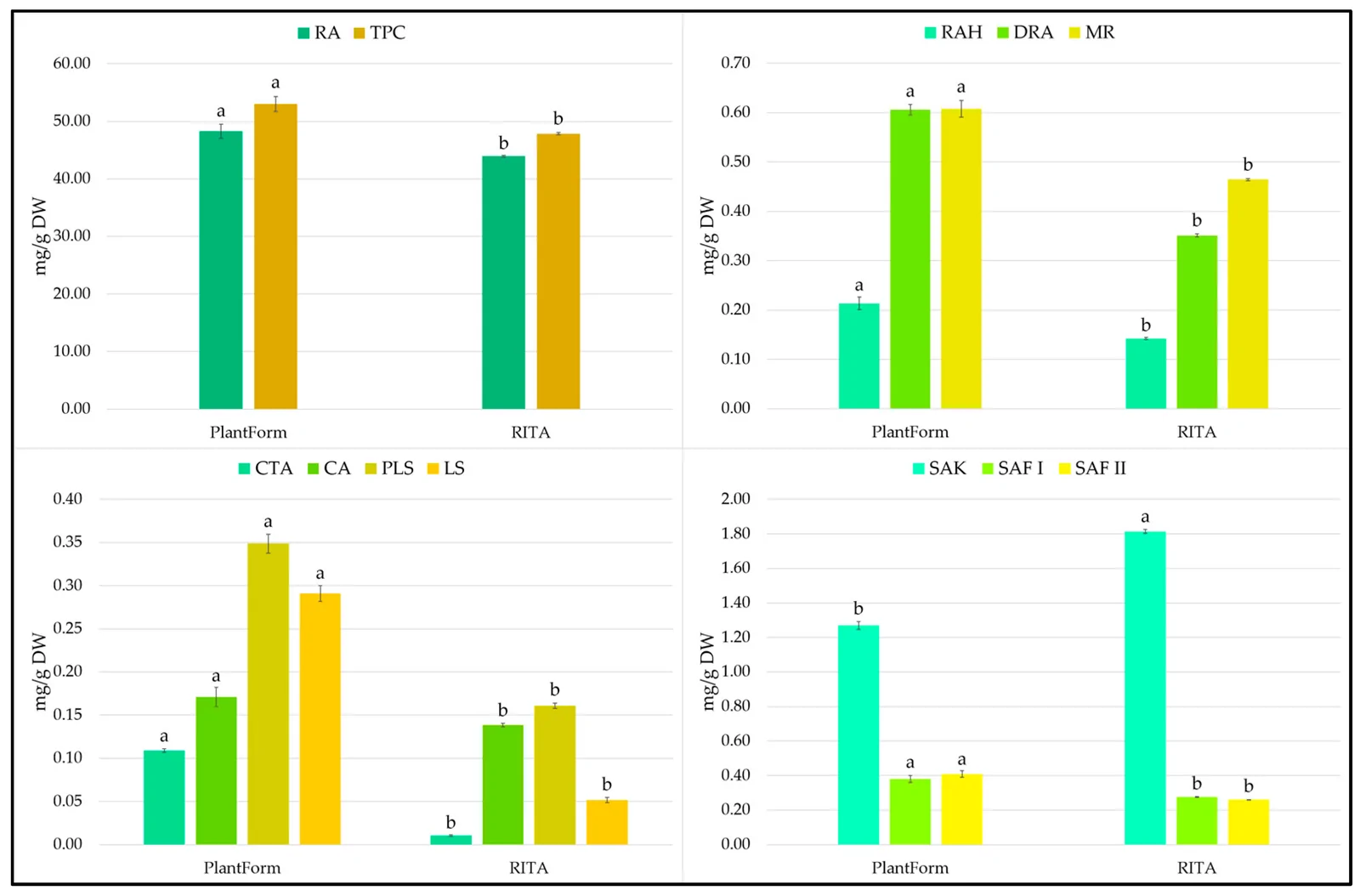
Secondary metabolite contents (mg/g DW) in *S. bulleyana* transformed shoot culture grown in bioreactor systems. The values represent the mean ± standard error of three independent experiments. Different letters for the same metabolite indicate significant differences between treatments at *p* < 0.05. CTA—caffeoyl-threonic acid isomer; CA—caffeic acid; PLS—protolithospermic acid isomer; RAH—rosmarinic acid hexoside; RA—rosmarinic acid; SAK—salvianolic acid K; LS—lithospermic acid isomer; DRA—dehydrorosmarinic acid; MR—methyl rosmarinate; SAF I—salvianolic acid F isomer I; SAF II—salvianolic acid F isomer II; TPC—total polyphenol content.

Shoots cultivated in the PlantForm™ bioreactor accumulated a higher total polyphenol (TPC) content, i.e., 53.0 mg/g DW, which was approximately 10% higher than in the RITA^®^ system (47.9 mg/g DW). A similar trend was observed for the predominant compound, RA, whose content amounted to 48.3 mg/g DW in PlantForm™ and 43.9 mg/g DW in RITA^®^ (Figure 2).

The PlantForm™ system achieved greater accumulation of the majority phenolic acids, viz. caffeoyl-threonic acid derivative (CTA), lithospermic acid isomer (LS), and protolithospermic acid (PLS) in *S. bulleyana* shoots; their respective contents were approximately 11-, 6-, and 2-fold higher compared to the RITA^®^ culture. Moreover, higher levels of several rosmarinic acid derivatives (RAH—rosmarinic acid hexoside; DRA—dehydrorosmarinic acid; MR—methyl rosmarinate), as well as caffeic acid (CA) and both salvianolic acid (SAF) isomers, were also recorded in PlantForm™ bioreactor shoots (Figure 2).

In contrast, the only compound that accumulated at a higher level in the RITA^®^ system was SAK, reaching 1.8 mg/g DW, i.e., approximately 43% higher than in the PlantForm™ culture (Figure 2).

While the PlantForm™ system achieved generally higher metabolite concentrations (mg/g DW), the RITA^®^ bioreactor sometimes demonstrated greater overall productivity, expressed per unit volume of medium (mg/L). The RITA^®^ system achieved higher TPC (755.1 mg/L) due to higher biomass growth dynamics and increased productivity of selected metabolites (mg/L), particularly RA (8% higher) and SAK (69%), compared to the PlantForm™ culture (Table 2).

### 2.2. Hairy Root Cultivations

**Figure 3:**
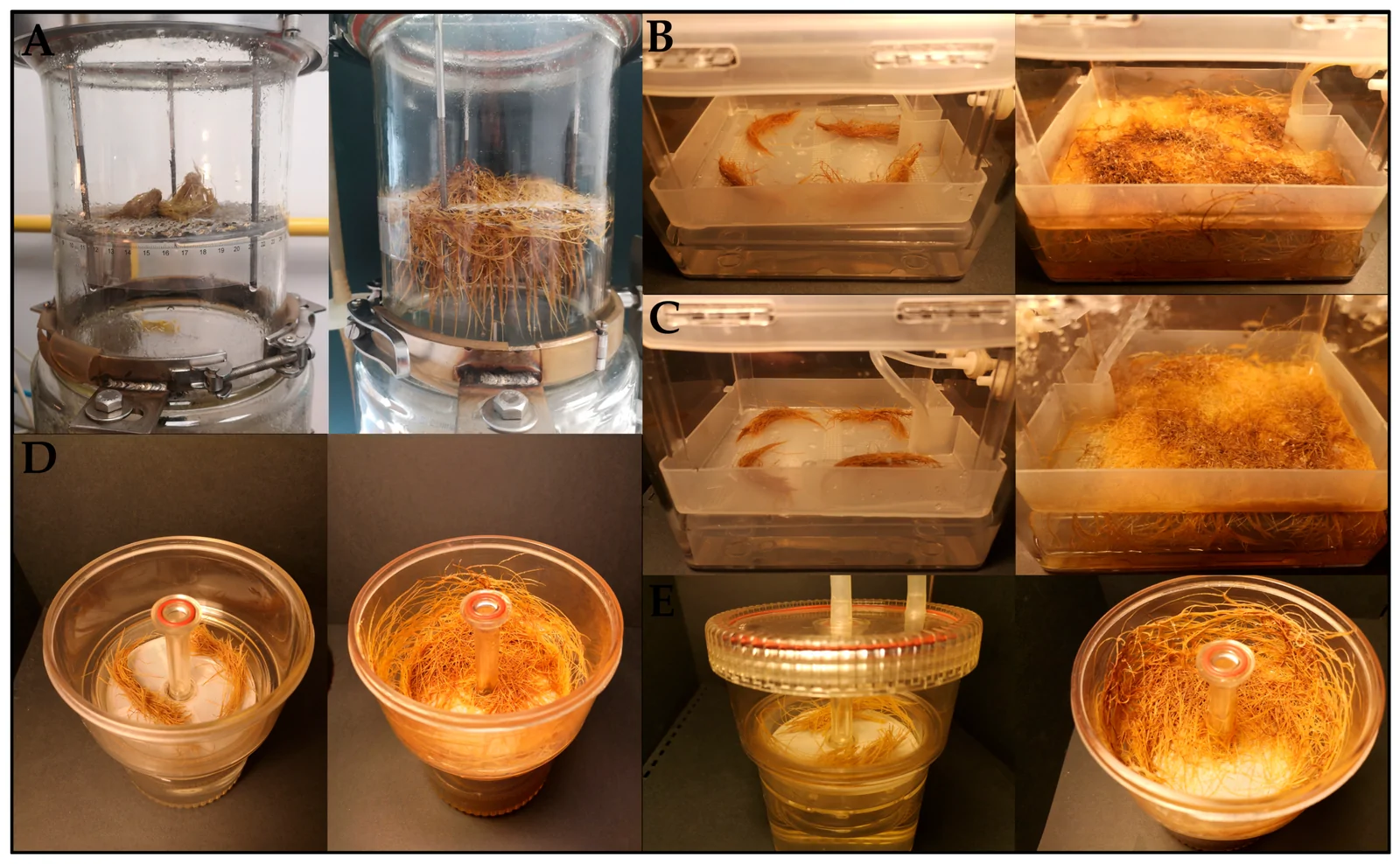
Hairy root culture of *S. bulleyana* cultivated in bioreactor systems: (**A**) in nutrient sprinkle bioreactor (inoculum, culture after 40 days); (**B**) PlantForm™ (3 min/90 min) (inoculum, culture after 40 days); (**C**) PlantForm™ (10 min/180 min) (inoculum, culture after 40 days); (**D**) RITA^®^ (3 min/90 min) (inoculum, culture after 40 days); (**E**) RITA^®^ (10 min/180 min) (inoculum, culture after 40 days).

The *S. bulleyana* hairy root culture was scaled up using two temporary immersion systems (PlantForm™ and RITA^®^) and a nutrient sprinkle bioreactor (Figure 3). The roots were cultivated for 40 days in liquid ½ SH medium supplemented with half concentration of vitamins and 3% (*w*/*v*) sucrose. Following this, biomass accumulation (FW and DW) and phenolic acid production were evaluated.

In all bioreactor systems, the hairy roots exhibited a typical phenotype characterized by intensive branching and the presence of numerous thin lateral roots. The biomass was light brown; however, after 40 days of cultivation, partial darkening of the biomass core regions was observed, especially in the PlantForm™ bioreactor (Figure 3).

The type of bioreactor significantly affected biomass accumulation in *S. bulleyana* hairy roots. The NSB achieved the highest absolute fresh (44.0 g FW) and dry (6.44 g DW) biomass values (Table 3), as well as the greatest productivity per unit volume of medium, achieving 88.0 g/L FW and 12.9 g/L DW. In addition, the NSB demonstrated the highest growth indices, reaching 12.2 for fresh weight and 11.2 for dry weight.

Among the temporary immersion systems (TISs), significantly higher fresh weight productivity was obtained in RITA^®^ (65.7–67.0 g/L) than in PlantForm™ (50.4–56.7 g/L), whereas dry weight productivity did not differ significantly between the two. The RITA^®^ system also tended to exhibit higher growth indices (11.0–11.3 for FW and 11.1–11.2 for DW) than PlantForm™ (8.2–9.4 for FW and 9.6–9.7 for DW), indicating more efficient biomass multiplication relative to the initial inoculum. However, immersion regime (3 min/90 min vs. 10 min/180 min) had no significant effect on biomass accumulation or growth index values within either TIS (Table 3).

After 40 days of cultivation, all hairy root variants were subjected to quantitative analysis. Both the type of bioreactor and immersion regime used in the temporary immersion system significantly influenced phenolic acid biosynthesis.

**Figure 4:**
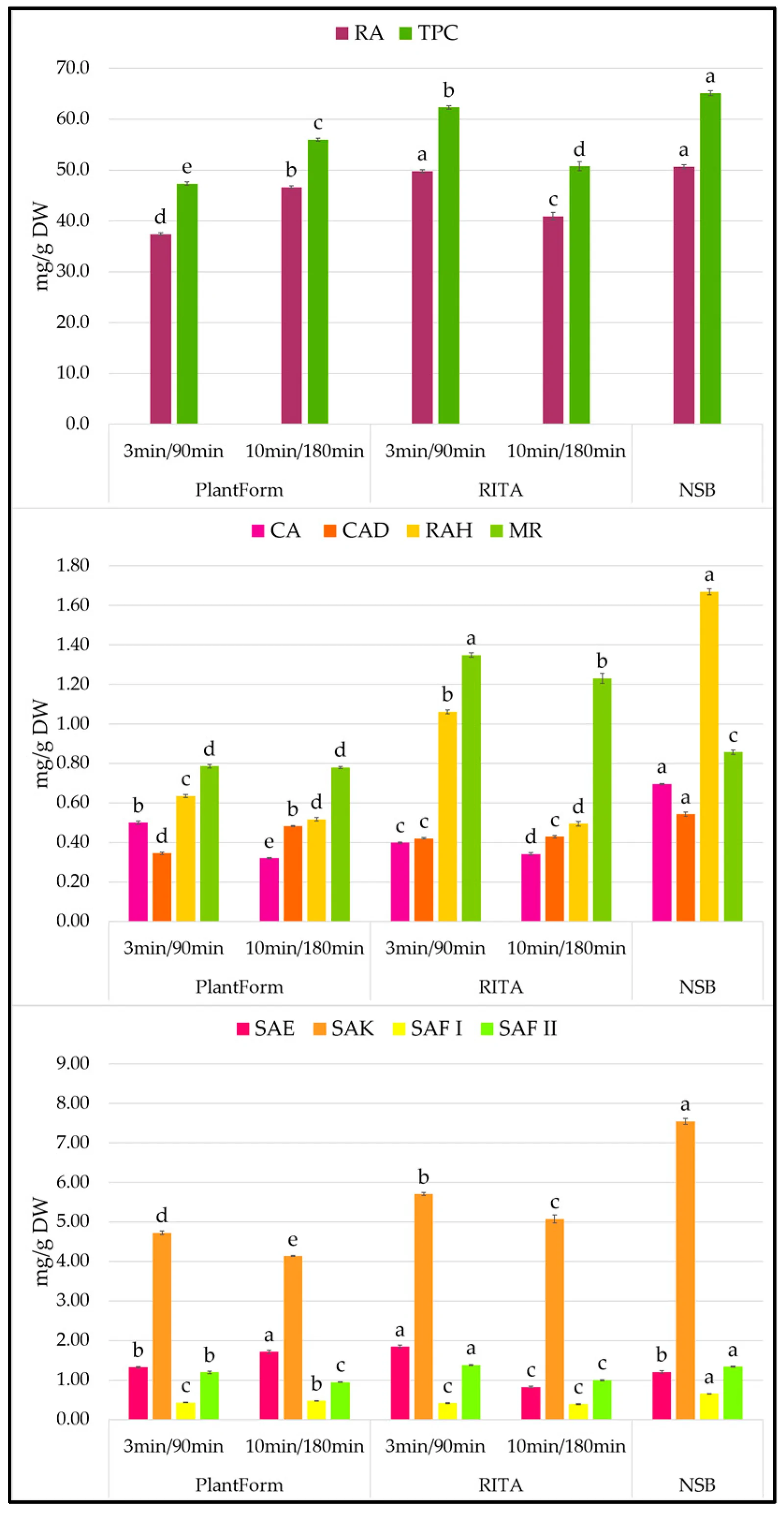
Secondary metabolite contents (mg/g DW) in *S. bulleyana* hairy root cultures grown in bioreactor systems. The values represent the mean ± standard error of three independent experiments. Different letters for the same metabolite indicate significant differences between treatments at *p* < 0.05. CA—caffeic acid; RAH—rosmarinic acid hexoside; SAE—salvianolic acid E; RA—rosmarinic acid; SAK—salvianolic acid K; CAD—caffeic acid derivative; MR—methyl rosmarinate; SAF I—salvianolic acid F isomer I; SAF II—salvianolic acid F isomer II; TPC—total polyphenol content.

Among the tested systems, the nutrient sprinkle bioreactor provided the most favorable conditions for polyphenol accumulation (Figure 4). The total polyphenol content reached 65.1 mg/g DW, which was significantly higher than all other treatments. RA was the dominant compound, accounting for approximately 78% of total phenolics (50.6 mg/g DW).

The NSB system also promoted the accumulation of other metabolites in *S. bulleyana* hairy roots, particularly rosmarinic acid hexoside (1.67 mg/g DW), whose content was 1.6- to 3.3-fold higher than in the other bioreactor systems (Figure 4). Additionally, the levels of caffeic acid, salvianolic acid K and salvianolic acid F isomer I were at least 30% higher in NSB compared to both PlantForm™ and RITA^®^ systems.

In the RITA^®^ bioreactor, the immersion regime significantly affected metabolite accumulation. Shorter and more frequent immersion (3 min every 90 min) resulted in higher TPC (62.3 mg/g DW) and RA content (49.8 m/g DW), i.e., approximately 20% higher than the longer and less frequent immersion regimens (Figure 4). This regimen also enhanced the accumulation of selected compounds, particularly RAH (1.06 mg/g DW) and SAE (1.84 mg/g DW), whose levels were more than two-fold higher compared to the alternative immersion schedule. In contrast, none of the metabolites analyzed in hairy roots achieved higher levels in the RITA^®^ bioreactor during longer immersion regimes (Figure 4).

In contrast, in the PlantForm™ bioreactor, 10 min immersion every 180 min was more favorable for phenolic acid accumulation. Under these conditions, TPC reached 56.0 mg/g DW, representing an 18% increase compared to the shorter immersion variant (3 min every 90 min). Similarly, RA content was higher (46.6 vs. 37.4 mg/g DW, respectively) (Figure 4). Longer immersion also promoted the accumulation of selected metabolites, including CAD, SAE, and SAF I, whose respective contents were approximately 40%, 30%, and 10% higher than in the shorter immersion variant. However, shorter immersion favored the accumulation of CA (+56%), RAH (+23%), SAK (+14%), and SAF II (+26%) in PlantForm™ system (Figure 4).

The NSB system was clearly the most efficient when expressing metabolite production per unit volume of medium: culture resulted in the accumulation of 28% to 95% more total phenolic compounds compared to the other systems. The superior productivity was primarily associated with elevated levels of RA (25–91% higher), as well as increased accumulation of CA (96–204%), RAH (94–353%), SAK (62–156%), CAD (56–122%), SAF I (91–114%), and SAF II (19–97%) (Table 4).

Overall, the NSB system provided the most favourable conditions for both biomass-associated metabolite accumulation and productivity with maximum TPC (837.9 mg/L) and RA (651.3 mg/L) levels (Table 4).

### 2.3. Effect of Methyl Jasmonate Treatment on Transformed Shoot and Hairy Root Cultures

Due to their high biomass productivity and phenolic compound accumulation, the nutrient sprinkle bioreactor and RITA^®^ system were selected for elicitation experiments in hairy root and transformed shoot cultures, respectively. In both cases, 100 μm methyl jasmonate (MeJA) was applied at the late growth stage of the culture cycle, and the effects were evaluated after three days of treatment. The corresponding growth and phytochemical characteristics of the non-elicited cultures are presented in Table 1, Table 2, Table 3 and Table 4 and Figure 2 and Figure 4, enabling direct comparison with the elicited cultures shown in Table 5 and Table 6. In both culture types, elicitation markedly stimulated phenylpropanoid metabolism, confirming that *S. bulleyana* cultures demonstrate high metabolic responsiveness under optimized bioreactor conditions.

While MeJA treatment did not inhibit biomass growth in the shoot cultures, it reduced growth by approximately 15% in the hairy roots (Table 5 and Table 6 vs. Table 1 and Table 3).

Elicitation markedly stimulated phenolic biosynthesis in both culture systems (Table 5 and Table 6). In hairy root cultures, RA accumulation increased from 50.6 to 73.0 mg/g DW, corresponding to a 45% increase relative to the control, while total polyphenol content increased from 65.1 to 85.9 mg/g DW (Table 6 vs. Figure 4). In transformed shoots, the elicitation response was even more pronounced, with RA content increasing from 43.9 to 71.6 mg/g DW, i.e., over 60% higher than untreated cultures (Table 5 vs. Figure 2). Total polyphenol content also increased from 47.9 to 76.9 mg/g DW. As a consequence, volumetric productivity reached 794.9 mg/L RA and 933.1 mg /L total polyphenols in hairy roots (Table 6), and 1164.2 mg/L RA and 1249.9 mg/L total polyphenols in transformed shoot cultures (Table 5). The relatively lower increase in productivity observed in the hairy roots compared with the shoots could be attributed to the partial inhibitory effect of MeJA on root biomass.

In addition to causing a pronounced increase in RA accumulation, MeJA also exerted a broader stimulatory effect on phenolic metabolism, particularly in transformed shoots. Elicitation caused a marked elevation in LS, SAK, SAF I, SAF II, and PLS levels and a decrease in MR and DRA (Table 5 vs. Figure 2). In hairy root cultures, the strongest response to the elicitor was observed for SAE, which exhibited than three-fold greater accumulation relative to the control (Table 6 vs. Figure 4). In contrast, the levels of several other major phenolic compounds remained unchanged or decreased. This indicates that MeJA preferentially redirected metabolic flux toward selected branches of phenylpropanoid metabolism rather than uniformly stimulating the accumulation of all detected metabolites.

## 3. Discussion

Biotechnological approaches to plant propagation and culture offer a number of advantages over traditional soil-based cultivation. Most importantly, they allow sustainable production of medicinal plant biomass and bioactive metabolites without exerting pressure on natural populations. In addition, bioreactor-based systems offer more controlled, reproducible, and scalable conditions for more precise cultivation [14]. However, the efficiency of a particular system strongly depends on species-specific characteristics, the type of propagated material, and the optimization of environmental parameters, including aeration, nutrient supply, and hydrodynamic conditions. Consequently, bioreactor configuration remains a critical factor determining biomass growth and metabolite productivity [12,13].

Our findings demonstrate that *S. bulleyana* can be successfully cultivated in different bioreactor systems. However, biomass accumulation and secondary metabolite production were found to be strongly dependent on both bioreactor type and operational regime. Clear differences were also observed between transformed shoot cultures and hairy roots, indicating distinct physiological responses to cultivation conditions. Such variability is to be expected, as the transition from shake-flask cultures to bioreactor systems substantially alters mass transfer properties and physicochemical conditions; these frequently affect both growth dynamics and secondary metabolism [20,24].

In the case of transformed shoots, TISs supported efficient biomass proliferation and enhanced phenolic compound production. This is in line with previous research indicating that TISs offer improved shoot growth and metabolite accumulation over continuously submerged cultures; this has been attributed to their use of periodic nutrient exposure combined with improved gas exchange and reduced hyperhydric stress [14,16]. Similar effects have been reported for, inter alia, *Myrtus communis*, *Schisandra chinensis*, and *Arnica montana* cultivated in PlantForm™ or RITA^®^ systems [25,26,27]. Similarly, the transformed shoot cultures of *S. bulleyana* in the present study exhibited high proliferation rates and phenolic acid accumulation in both bioreactors, exceeding those obtained in solid-medium cultures [9]. Rosmarinic acid remained the dominant metabolite, and its accumulation was substantially higher than previously reported for field-grown plants [7], confirming the high biosynthetic potential of the system.

Despite the generally positive response, the two TISs differed in their effects on biomass growth and metabolite accumulation. While the RITA^®^ bioreactor promoted more intensive biomass proliferation and higher volumetric productivity, these effects were accompanied by some hyperhydricity. Excessive tissue hydration is known to disrupt cellular organization and negatively affect secondary metabolism, which may explain the lower phenolic compound accumulation observed in the system. The relationships between hyperhydricity and reduced polyphenol biosynthesis were previously reported in *Castilleja tenuiflora* and *Salvia officinalis* cultures [20,28]. In contrast, the PlantForm™ system provided more stable morphogenetic conditions and limited hyperhydricity, resulting in higher phenolic accumulation on a dry weight basis. Comparable observations were reported for *Centella asiatica*, where PlantForm™ cultivation enhanced the production of phenolic acids, and other secondary metabolites, more effectively than RITA^®^ [29].

A markedly different response was observed in hairy root cultures. Although RA remained the predominant metabolite in all tested systems, the temporary immersion bioreactors proved considerably less effective for the cultivation of hairy roots than for transformed shoots. In contrast, the nutrient sprinkle bioreactor demonstrated significantly greater accumulation of RA and its derivatives, indicating that the system provides more favourable conditions for hairy root metabolism. Similarly, nutrient sprinkle systems were found to enhance phenolic compound production in *Agastache rugosa* and *Rhaponticum carthamoides* hairy root cultures compared to temporary immersion bioreactors [21,22].

The different responses demonstrated by the shoot and hairy root cultures likely result from their specific tissue organization and oxygen transfer requirements. Temporary immersion systems create favourable conditions for shoot cultures because alternating immersion and aeration phases ensure efficient nutrient uptake while maintaining adequate gas exchange. In contrast, hairy roots can form dense and highly branched biomass aggregates, which may substantially restrict internal oxygen diffusion. This oxygen availability may be further limited during immersion, when the liquid medium fills the interstitial spaces within the root. Such limitations are considered one of the major factors restricting hairy root performance in liquid-based bioreactor systems [10,30,31].

Interestingly, in both TISs, immersion frequency and duration had only a limited effect on hairy root biomass growth, suggesting that such changes could not overcome the intrinsic diffusion limitations associated with dense root aggregates. Nevertheless, these conditions significantly influenced secondary metabolite accumulation, and clear differences were observed between RITA^®^ and PlantForm™ systems. In RITA^®^, higher metabolite production was associated with shorter and more frequent immersion cycles; in PlantForm™, improved accumulation was observed under longer and less frequent immersion. These differences may reflect the specific aeration efficiency and hydrodynamic conditions of the two systems, which could differentially influence transient oxygen fluctuations and stress-related metabolic responses. Indeed, oxygen availability and temporary stress conditions have been found to strongly affect phenylpropanoid metabolism and stimulate secondary metabolite biosynthesis in plant in vitro cultures [32,33].

The superior performance observed in the NSB is consistent with the hypothesis that oxygen transfer plays a major role in hairy root cultivation. In this system, the roots remain predominantly exposed to the gas phase and are intermittently supplied with nutrients as fine droplets, which improves oxygen availability and reduces diffusion limitations within the biomass. Such conditions are particularly advantageous for highly branched root systems characterized by elevated oxygen demand [34,35]. The alternating spray–aeration regime likely enabled more balanced growth conditions, resulting in enhanced biomass productivity and increased accumulation of RA derivatives.

Although the nutrient sprinkle bioreactor showed the best performance among the systems evaluated in this study, several methodological aspects should be considered when interpreting direct comparisons among the tested cultivation systems. It should be noted that inoculum size and configuration were adjusted to the working volume, cultivation geometry, and manufacturer-recommended operating conditions of each bioreactor rather than standardized across all systems. This approach was necessary to maintain comparable biomass density and provide adequate space for culture development within the structural constraints of each cultivation vessel. Biomass growth and metabolite production were therefore evaluated using normalized parameters, including growth indices, metabolite concentrations (mg/g DW), and volumetric productivity (mg/L), thereby minimizing the influence of differences in inoculum mass and medium volume. Nevertheless, because inoculum configuration was inherently linked to the design and operating principles of each cultivation system, direct comparisons among the tested bioreactors should be interpreted with appropriate caution.

While the *S. bulleyana* bioreactor cultures achieved high rosmarinic acid productivity, further enhancement of polyphenol accumulation remains desirable. One of the most effective approaches for stimulating secondary metabolism in plant in vitro systems is elicitation with methyl jasmonate (MeJA). Both transformed shoots and hairy roots demonstrated significant responses to MeJA elicitation, which confirms the high metabolic plasticity of *S. bulleyana* cultures. It also confirms that the phenylpropanoid metabolism can be further stimulated under optimized conditions. Jasmonates are key signaling molecules involved in plant defense responses. They are known to activate secondary metabolism through the induction of genes associated with the phenylpropanoid pathway and rosmarinic acid biosynthesis [36]. Methyl jasmonate treatment has previously been reported to enhance phenolic acid accumulation in several Lamiaceae species, including *Salvia miltiorrhiza* and *Agastache rugosa* cultures [21,37].

Interestingly, the elicitation response differed between transformed shoots and hairy roots, indicating distinct physiological and metabolic adaptations between the two. In hairy roots, the increase in metabolite accumulation was accompanied by partial growth inhibition, which is frequently observed following jasmonate treatment. This effect has been attributed to the reallocation of cellular resources from biomass formation to the defense-related secondary metabolism. In contrast, transformed shoots maintained high growth while exhibiting pronounced stimulation of phenolic compound biosynthesis. These observations suggest that the final elicitation outcome depends not only on elicitor activity, but also on interactions between culture type, growth dynamics, and bioreactor microenvironment.

Collectively, our findings indicate that combining optimized bioreactor cultivation with elicitor-based metabolic stimulation represents an effective strategy for maximizing RA and total polyphenol productivity in *S. bulleyana* culture. Higher levels of RA were obtained compared to previous data obtained for both shoot and hairy root cultures cultivated in bioreactor systems. Under the optimized conditions with the MeJA elicitor, maximum RA productivity was 1164.2 mg/L in transformed shoots cultured in the RITA^®^ bioreactor and 794.9 mg/L in hairy roots maintained in the nutrient sprinkle bioreactor. These values exceed most reports available for *Salvia* species and other representatives of the Lamiaceae family cultivated in vitro under scaling-up conditions. For example, *S. viridis* shoot cultures grown in TIS achieved accumulated 80 mg/L RA [17], while *S. officinalis* hairy roots cultivated in a sprinkle bioreactor achieved 477.1 mg/L [20]. Similarly, in hairy root cultures of *Agastache rugosa*, the highest phenolic compound productivity was achieved in a nutrient sprinkle bioreactor (79.7 mg/L); this value compares favourably with 71.6 mg/L in the RITA^®^ system and only 11.1 mg/L in the PlantForm™ bioreactor [21].

Although bioreactor cultivation generally enhances rosmarinic acid biosynthesis in Lamiaceae species, the productivity achieved in *S. bulleyana* indicates particularly high biosynthetic potential. It also highlights the suitability of this species for scalable production of phenolic compounds.

## 4. Materials and Methods

### 4.1. Plant Material

The transformed shoot culture of *S. bulleyana* was obtained via spontaneous regeneration from transformed roots, as described previously [9]. The shoots were maintained on solid MS medium [38] supplemented with 0.7% (*w*/*v*) agar, 0.1 mg/L indole-3-acetic acid (IAA), and 1.0 mg/L 6-benzylaminopurine riboside (BAPR). Subcultures were performed every 35 days. The cultures were grown in a controlled growth chamber at 26 °C, under a 16 h photoperiod, light intensity of 40 μmol /m^2^ s^1^, relative humidity of 80–90%.

Hairy root cultures were established via transformation with *Rhizobium rhizogenes* strain A4, according to Krzemińska et al. (2022) [11]. The roots were cultivated in 80 mL of liquid SH medium [39] containing half-strength macro- and microelements, and vitamins (½SH, ½V). The cultures were maintained on a rotary shaker at 70 rpm, at 26 °C, in complete darkness. Subculturing was performed at 40-day intervals. The culture conditions were optimized as described previously [11].

### 4.2. Bioreactor Systems

Three types of bioreactor systems were used in the experiments: two commercially available temporary immersion systems (TIS), i.e., PlantForm™ (PlantForm™, Hjärup, Sweden) [40] and RITA^®^ (Récipient à Immersion Temporaire Automatique, VITROPIC, Saint-Mathieu de Tréviers, France) [41], and a nutrient sprinkle bioreactor (NSB) described by Szymańska et al. (2025) [42].

The PlantForm™ system was operated with 500 mL of liquid medium and aerated using an Optima pump (Hagen, Baie-d’Urfé, QC, Canada) at a volumetric flow rate of 0.6 m^3^/h. The RITA^®^ bioreactor contained 250 mL of medium and was supplied with air using a DT4.4 pump (Becker, Wuppertal, Germany) at a flow rate of 4.2 m^3^/h. In the two TISs, immersion was performed for 3 min every 90 min for shoot cultures, and for 3 min every 90 min or 10 min every 180 min for root culture. Detailed technical specifications and operational principles of the systems are available from the manufacturers (PlantForm™, RITA^®^).

The NSB (total volume 5 L) was operated with 0.5 L of liquid medium. The medium was circulated using a peristaltic pump (CMF 10, Chemap, Zurich, Switzerland) and a spraying nozzle, according to the following cycle: 1 min medium delivery followed by a 1 min break.

#### 4.2.1. Transformed Shoot Cultivation

The transformed shoot culture of *S. bulleyana* was cultivated in PlantForm™ and RITA^®^ bioreactors using liquid MS medium; the medium was supplemented with 0.1 mg/L indole-3-acetic acid (IAA) and 1.0 mg/L 6-benzylaminopurine riboside (BAPR). The inoculum consisted of shoot tips (approximately 1 cm in length): 20 explants for PlantForm™ (FW 1.12 ± 0.081 g; DW 0.102 ± 0.007 g) and 10 explants for RITA^®^ (FW 0.558 ± 0.041 g; DW 0.051 ± 0.004 g).

Cultures were maintained under a 16 h photoperiod with a light intensity of 40 μmol/m^2^ s at 26 °C for five weeks. The experiment was repeated three times.

At the end of the experiment, fresh weight (FW), dry weight (DW), growth index (GI), shoot length, multiplication rate, and morphological characteristics were determined. FW was measured after removal of excess medium, and DW after lyophilisation. Multiplication rate was expressed as a mean value of the sum of developed shoots and buds per explant. Growth index was calculated as follows FW(DW) − FW_i_(DW_i_)/FW_i_(DW_i_), where FW_i_(DW_i_) was the fresh (dry) weight of inoculum and FW(DW) was the fresh (dry) weight after growth cycle.

#### 4.2.2. Hairy Root Cultivation

Hairy root cultures were cultivated in PlantForm™, RITA^®^, and NSB using liquid ½SH medium with reduced macro- and microelement and vitamins concentrations (½V).

The inoculum size depended on the system used:

- PlantForm™: four root fragments (total FW 2.729 ± 0.113 g; total DW 0.430 ± 0.018 g);
- RITA^®^: two root fragments (total FW 1.364 ± 0.056 g; total DW 0.215 g ± 0.009 g);
- NSB: one root fragment (FW 3.345 ± 0.044 g; DW 0.527 ± 0.007 g).

Cultures were maintained in the dark at 26 °C for 40 days. The experiment was repeated three times. After the cultivation period, biomass accumulation (FW and DW) and growth index (GI) were evaluated. GI values were calculated as for shoot culture.

### 4.3. Phytochemical Analysis

Lyophilized and micronized plant material was extracted once with 30 mL and then twice with 15 mL of 80% methanol using ultrasonic desintegrator at 40 °C for 15 min. For shoot extract preparation, the micronized plant material was initially treated with 15 mL of chloroform to remove ballast compounds, such as chlorophyll. Finally the extracts were combined and evaporated to dryness. Before analysis, dry extracts were dissolved in 10 mL of 80% methanol and filtered using 0.22 μm nylon filters. Hydromethanolic extracts were analysed as described previously [9,11].

Briefly, quantitative analysis of polyphenolic compounds was performed using high-performance liquid chromatography (HPLC) on an Elite LaChrom Hitachi system (Merck, Darmstadt, Germany) equipped with an Ascentis^®^ Express C18 column (7.5 cm × 4.6 mm, 2.7 μm; Supelco, Bellefonte, PA, USA). The mobile phase consisted of 0.5% (*w*/*w*) aqueous orthophosphoric acid (solvent A) and acetonitrile (solvent B), applied in a gradient elution program: 0.0–1.0 min, 5% B (isocratic elution); 1.0–16.0 min, 5–30% B (*v*/*v*, linear gradient); 16.0–25.0 min, 30–50% B; 26.0–27.0 min 50–5% B; 27.0–32.0 min, 5% B (equilibration). Detection was carried out at 325 nm. Any compounds were identified by comparing retention times and UV spectra with authentic standards. Quantification was performed using external calibration curves. The content of individual compounds was expressed as mg/g DW of plant material. Total polyphenol content was calculated as a sum of all quantified compounds.

### 4.4. Methyl Jasmonate Treatment

The cultures demonstrating the highest rosmarinic acid and total polyphenol levels were included in further experiments to determine the effect of elicitation; these included hairy root cultures cultivated in the NSB and transformed shoot culture in RITA^®^. Methyl jasmonate (MeJA) was added to a final concentration of 100 μm on day 37 of the culture cycle to root culture, and on day 32 to shoot culture. The elicitor stock solution was sterilized by filtration (0.22 μm) prior to addition to the culture medium. Cultures were harvested three days after treatment. The elicitation parameters used in the experiment were previously developed based on hairy root culture carried out in flasks [8]. During the elicitation phase, the bioreactors were operated under standard mode: 1 min spraying/1 min break for NSB, and 3 min immersion every 90 min for RITA^®^, ensuring homogeneous exposure of the biomass to the elicitor supplemented-medium.

Biomass accumulation and secondary metabolite production were then analyzed as described for previous studies.

### 4.5. Statistical Analysis

All experiments were performed in triplicate, and the results presented as the mean ± standard error (SE). The significance of the differences between treatments was evaluated by one-way analysis of variance (ANOVA). When significant effects were detected, Tukey’s post hoc test was applied for comparisons between means. Differences were considered statistically significant at *p* < 0.05. All statistical analyses were performed using Statistica 13.1 PL software (StatSoft Inc., Kraków, Poland).

## 5. Conclusions

The efficiency of *Salvia bulleyana* in vitro cultures is strongly dependent on bioreactor configuration and operational regime, which critically affect biomass growth, metabolite accumulation, and process productivity. The nutrient sprinkle bioreactor proved to be the most effective system for hairy root cultures, whereas temporary immersion systems were more suitable for transformed shoots, although their performance was influenced by immersion parameters and hyperhydricity. Hence, for each culture configuration, the conditions require organ-specific optimization for efficient biomass propagation and phenolic compound production.

The addition of methyl jasmonate elicitation to optimized bioreactor conditions further enhanced secondary metabolite biosynthesis, resulting in rosmarinic acid productivity exceeding 1000 mg/L and total polyphenol productivity over 1200 mg/L. These values substantially exceed those previously reported for comparable Lamiaceae in vitro culture systems and confirm the high biosynthetic potential of *S. bulleyana*. Collectively, the study demonstrates that the combination of tailored bioreactor strategies and elicitor-based metabolic stimulation provides an effective platform for scalable production of high-value phenolic compounds. Our study provides a practical framework for process optimization and supports the further development of commercially viable plant-based biotechnological production systems.

## Figures and Tables

**Table 1:** The growth parameters of *S. bulleyana* transformed shoot culture grown in bioreactor systems after 5 weeks.

**Bioreactor Type**	**FW (g)**	**DW (g)**	**FW (g/L)**	**DW (g/L)**	**Growth Index**	
					**FW**	**DW**
PlantForm™	69.2 ± 3.71 a	6.67 ± 0.13 a	138.3 ± 7.4 b	13.3 ± 0.25 b	61.0 ± 3.32 b	64.4 ± 1.23 b
RITA^®^	47.6 ± 0.61 b	3.95 ± 0.06 b	190.5 ± 2.4 a	15.8 ± 0.22 a	84.4 ± 1.09 a	76.4 ± 1.08 a
	**Main Shoot Length (cm)**		**Regenerated Shoot Length (cm)**		**Multiplication Rate**	**Root Length (cm)**
PlantForm™	2.54 ± 0.15 b		1.15 ± 0.14 a		5.26 ± 0.52 b	0.95 ± 0.12 a
RITA^®^	4.08 ± 0.22 a		1.25 ± 0.23 a		7.32 ± 0.91 a	0.89 ± 0.14 a

The values represent the mean ± standard error of three independent experiments. Different letters for the same parameter indicate significant differences between treatments at *p* < 0.05.

**Table 2:** Productivity (mg/L) of bioactive metabolites in *S. bulleyana* transformed shoot culture grown in bioreactor systems.

	PlantForm™	RITA^®^
CTA	1.45 ± 0.02 a	0.161 ± 0.004 b
CA	2.28 ± 0.15 a	2.18 ± 0.02 a
PLS	4.65 ± 0.15 a	2.54 ± 0.04 b
RAH	2.85 ± 0.18 a	2.25 ± 0.03 b
RA	643.6 ± 16.4 b	693.5 ± 2.54 a
SAK	16.9 ± 0.31 b	28.6 ± 0.18 a
LS	3.90 ± 0.12 a	0.813 ± 0.04 b
DRA	8.08 ± 0.15 a	5.54 ± 0.05 b
MR	8.10 ± 0.23 a	7.33 ± 0.03 b
SAF I	5.08 ± 0.27 a	4.37 ± 0.04 b
SAF II	5.45 ± 0.26 a	4.12 ± 0.02 b
TPC	707.0 ± 17.4 b	755.1 ± 2.76 a

CTA—caffeoyl-threonic acid isomer; CA—caffeic acid; PLS—protolithospermic acid isomer; RAH—rosmarinic acid hexoside; RA—rosmarinic acid; SAK—salvianolic acid K; LS—lithospermic acid isomer; DRA—dehydrorosmarinic acid; MR—methyl rosmarinate; SAF I—salvianolic acid F isomer I; SAF II—salvianolic acid F isomer II; TPC—total polyphenol content. The values represent the mean ± standard error of three independent experiments. Different letters for the same metabolite indicate significant differences between treatments at *p* < 0.05.

**Table 3:** The growth parameters of *S. bulleyana* hairy root culture grown in bioreactor systems after 40 days.

Bioreactor Type		FW (g)	DW (g)	FW (g/L)	DW (g/L)	Growth Index	
						FW	DW
PlantForm™	3 min/90 min	25.2 ± 1.17 b	4.55 ± 0.26 b	50.4 ± 2.35 c	9.1 ± 0.51 b	8.2 ± 0.43 b	9.6 ± 0.59 a
	10 min/180 min	28.3 ± 1.35 b	4.60 ± 0.17 b	56.7 ± 2.69 c	9.2 ± 0.35 b	9.4 ± 0.49 b	9.7 ± 0.41 a
RITA^®^	3 min/90 min	16.8 ± 1.48 c	2.62 ± 0.15 c	67.0 ± 5.90 b	10.5 ± 0.61 b	11.3 ± 1.08 b	11.2 ± 0.70 a
	10 min/180 min	16.4 ± 1.66 c	2.61 ± 0.14 c	65.7 ± 6.64 b	10.4 ± 0.56 b	11.0 ± 1.22 b	11.1 ± 0.65 a
NSB		44.0 ± 0.17 a	6.44 ± 0.03 a	88.0 ± 0.35 a	12.9 ± 0.05 a	12.2 ± 0.05 a	11.2 ± 0.05 a

The values represent the mean ± standard error of three independent experiments. Different letters for the same parameter indicate significant differences between treatments at *p* < 0.05.

**Table 4:** Productivity (mg/L) of bioactive metabolites in *S. bulleyana* hairy root culture grown in bioreactor systems.

	PlantForm™ 3 min/90 min	PlantForm™ 10 min/180 min	RITA^®^ 3 min/90 min	RITA^®^ 10 min/180 min	NSB
CA	5.56 ± 0.068 b	2.95 ± 0.02 e	4.19 ± 0.04 c	3.56 ± 0.06 d	8.96 ± 0.04 a
RAH	5.78 ± 0.07 c	4.75 ± 0.09 d	11.1 ± 0.12 b	5.15 ± 0.12 d	21.5 ± 0.20 a
SAE	12.0 ± 0.18 c	15.8 ± 0.41 b	19.3 ± 0.37 a	8.56 ± 0.17 d	15.5 ± 0.45 b
RA	340.3 ± 2.29 d	428.4 ± 2.20 c	522.4 ± 3.36 b	425.7 ± 7.84 c	651.3 ± 4.71 a
SAK	43.0 ± 0.39 d	38.0 ± 0.11 e	59.9 ± 0.42 b	52.8 ± 1.00 c	97.1 ± 1.02 a
CAD	3.15 ± 0.06 c	4.43 ± 0.03 b	4.42 ± 0.03 b	4.47 ± 0.07 b	6.99 ± 0.14 a
MR	7.16 ± 0.08 d	7.17 ± 0.04 d	14.2 ± 0.12 a	12.8 ± 0.28 b	11.0 ± 0.15 c
SAF I	3.89 ± 0.07 c	4.32 ± 0.05 bc	4.35 ± 0.12 b	4.03 ± 0.13 bc	8.33 ± 0.15 a
SAF II	10.9 ± 0.28 c	8.73 ± 0.05 d	14.4 ± 0.10 b	10.3 ± 0.08 c	17.2 ± 0.14 a
TPC	430.7 ± 2.85 d	514.6 ± 2.70 c	654.4 ± 4.01 b	527.4 ± 9.33 c	837.9 ± 6.41 a

CA—caffeic acid; RAH—rosmarinic acid hexoside; SAE—salvianolic acid E; RA—rosmarinic acid; SAK—salvianolic acid K; CAD—caffeic acid derivative; MR—methyl rosmarinate; SAF I—salvianolic acid F isomer I; SAF II—salvianolic acid F isomer II; TPC—total polyphenol content. The values represent the mean ± standard error of three independent experiments. Different letters for the same metabolite indicate significant differences between treatments at *p* < 0.05.

**Table 5:** Growth parameters (GI and g/L), bioactive metabolite accumulation (mg/g DW), and productivity (mg/L) in *S. bulleyana* transformed shoot culture grown in bioreactor systems and elicited with 100 μm MeJA.

	**Growth Index**	**g/L**
FW	76.3 ± 3.31	172.4 ± 7.38
DW	78.7 ± 2.37	16.3 ± 0.48
	**mg/g DW**	**mg/L**
CTA	0.130 ± 0.002	2.11 ± 0.04
CA	0.080 ± 0.002	1.25 ± 0.04
PLS	0.291 ± 0.006	4.73 ± 0.10
RAH	0.114 ± 0.003	1.85 ± 0.06
RA	71.6 ± 0.760	1164.2 ± 12.4
SAK	0.998 ± 0.066	16.2 ± 1.07
LS	0.129 ± 0.007	1.99 ± 0.16
DRA	1.61 ± 0.242	26.3 ± 3.94
MR	0.715 ± 0.020	11.6 ± 0.33
SAF I	0.414 ± 0.011	6.73 ± 0.18
SAF II	0.626 ± 0.026	10.2 ± 0.43
TPC	76.7 ± 1.03	1249.9 ± 16.70

CTA—caffeoyl-threonic acid isomer; CA—caffeic acid; PLS—protolithospermic acid isomer; RAH—rosmarinic acid hexoside; RA—rosmarinic acid; SAK—salvianolic acid K; LS—lithospermic acid; DRA—dehydrorosmarinic acid; MR—methyl rosmarinate; SAF I—salvianolic acid F isomer I; SAF II—salvianolic acid F isomer II; TPC—total polyphenol content. The values represent the mean ± standard error of three independent experiments.

**Table 6:** Growth parameters (GI and g/L), bioactive metabolite accumulation (mg/g DW), and productivity (mg/L) in *S. bulleyana* hairy root culture grown in bioreactor systems and elicited with 100 μm MeJA.

	**Growth Index**	**g/L**
FW	11.9 ± 0.24	86.1 ± 1.63
DW	9.30 ± 0.97	10.9 ± 1.03
	**mg/g DW**	**mg/L**
CA	0.426 ± 0.005	4.62 ± 0.06
RAH	1.15 ± 0.021	12.4 ± 0.23
SAE	4.08 ± 0.070	44.3 ± 0.76
RA	73.2 ± 1.15	794.9 ± 12.4
SAK	4.29 ± 0.078	46.6 ± 0.85
CAD	0.376 ± 0.006	4.09 ± 0.07
MR	0.990 ± 0.040	10.8 ± 0.44
SAF I	0.471 ± 0.009	5.12 ± 0.09
SAF II	0.941 ± 0.014	10.2 ± 0.15
TPC	85.9 ± 1.340	933.1 ± 14.60

CA—caffeic acid; RAH—rosmarinic acid hexoside; SAE—salvianolic acid E; RA—rosmarinic acid; SAK—salvianolic acid K; CAD—caffeic acid derivative; MR—methyl rosmarinate; SAF I—salvianolic acid F isomer I; SAF II—salvianolic acid F isomer II; TPC—total polyphenol content. The values represent the mean ± standard error of three independent experiments.

## Data Availability

The original contributions presented in this study are included in the article. Further inquiries can be directed to the corresponding author.
